# Prevalence of depression and anxiety among male patients with COVID-19 in Lebsayyer Field Hospital, Qatar

**DOI:** 10.5339/qmj.2021.68

**Published:** 2021-12-01

**Authors:** Nashwan Zainal Deen, Amr Al-Sharafi, Mohamed Abdalla, Mohammed Mushtaha, Ahmad Mohamed, Sana Saleem, Yazan Nofal, Mohamed Adil Shah Khoodoruth, Abdulla Al-Naimi

**Affiliations:** ^1^Family Medicine Department, Hamad Medical Corporation, Qatar E-mail: Ndeen@hamad.qa; ^2^Urology Department, Hamad Medical Corporation, Qatar; ^3^Anesthesiology Department, Hamad Medical Corporation, Qatar; ^4^Neurology Department, Hamad Medical Corporation, Qatar; ^5^Psychiatry Department, Hamad Medical Corporation, Qatar

**Keywords:** COVID-19, pandemic, depression, anxiety, prevalence, Qatar

## Abstract

Background: Depression and anxiety are major health problems found to be associated with various conditions. COVID-19 is a global pandemic that has a substantial effect on the worldwide population. This study aimed to assess the prevalence of depression and anxiety among male patients with COVID-19 and explore their relationship with participants’ characteristics.

Methods: This cross-sectional study was conducted among expatriate male patients with COVID-19 admitted to Lebsayyer Field Hospital in Qatar with mild COVID-19 (according to World Health Organization classification) from July till August 2020. The sample size was calculated using Cochran's formula based on disease prevalence. All eligible patients were invited to participate until reaching 400 participants, who then completed an anonymous survey of sociodemographic questions, Patient Health Questionnaire-9, and Generalized Anxiety Disorder-7 questionnaire, which are validated screening tools for depression and anxiety, respectively.

Results: Of the 400 participants, 148 (37.0%) and 77 (19.3%) reported depressive and anxiety symptoms, respectively. Depression was more prevalent among those 40–49 years old (*p* = 0.029), while anxiety was more prevalent among people aged  ≥ 50 (*p* = 0.456). Both depression (*p* = 0.009) and anxiety (*p* = 0.042) were more prevalent among Bangladeshi, followed by Filipino participants. Depression was more prevalent among those with the highest income (> 15,000 QR; *p* = 0.004), in contrast to anxiety, which was more prevalent among those with the lowest monthly income ( <  2,000 QR; *p* = 0.039).

Conclusion: The prevalence of depressive and anxiety symptoms is high among the study participants. Associated factors identified by the study were unsteady income, poor self-rated health, living with family, Southeast Asian ethnicity, and age group of 40–49 years.

## Introduction

COVID-19 is an on-going global pandemic that was initially notified as a cluster of cases of “viral pneumonia of unknown cause” identified in Wuhan, China, in December 2019 and subsequently determined to be caused by severe acute respiratory syndrome coronavirus-2 (SARS-CoV-2).^1^ SARS-CoV-2 is a highly contagious and sometimes fatal virus rapidly spreading globally by human-to-human transmission via droplets or by indirect contact with contaminated objects.^2^ The outbreak was declared a public-health emergency of international concern in January 2020 and a pandemic in March 2020 by the World Health Organization.^3^

Qatar is a gulf state with a population of 2.8 million in 2020^4^, of which >85% were expatriates. Qatar recorded its first COVID-19 case on February 29, 2020^5^, and the numbers have been increasing and reached 227,055 by August 05, 2021.^6^ Since March 30, 2020, Hamad Medical Corporation (HMC) announced the designation of Hazm Mebaireek General Hospital as a COVID-19 treatment facility to provide high-quality care for patients with COVID-19.^7^ On April 09, 2020, two primary health centers were designated for screening, testing, and quarantine for suspected COVID-19 cases.^8^ Soon after, many new hospitals –including Lebsayyer Field Hospital (LFH) –were launched to provide care to patients with COVID-19. LFH is a temporary hospital previously used by the military and converted into a healthcare facility dedicated to receiving patients who were clinically improving from higher-level hospitals as well as patients with noncritically ill status in the community. LFH is a 504-bed facility established to admit blue-collar expatriate male workers, where every patient is admitted to a single en suite room. It provides them with disease monitoring, medical care, and social activities.

Widespread outbreaks of infectious diseases are not only associated with physical illness but also psychological distress and symptoms of mental disorders.^9^ Depression and anxiety are the most prevalent mental disorders worldwide. Depression characteristics include low mood or loss of interest and can cause significant difficulties in daily life.^10^ Anxiety is defined as excessive, out of proportion, and difficult-to-control worry and distress upon dealing with life events. Depression and anxiety often coexist. Each can negatively affect the natural history and outcomes of the other, increasing morbidity and mortality and accounting for substantial healthcare costs.^11^ Medical illnesses are associated with higher prevalence of depression and anxiety, and there are meaningful connections between viral diseases and depression and anxiety.^12^

The COVID-19 pandemic elicits stress due to fear of loss of employment, financial insecurity, social isolation, stigma, and death.^13^ Cuiyan et al. examined the initial psychological responses of the Chinese public to the COVID-19 outbreak using an online survey. They found that 16.5% of the respondents reported moderate-to-severe depressive symptoms, and 28.8% reported moderate-to-severe anxiety symptoms.^14^ Lei et al. also showed that the prevalence of anxiety and depression among individuals affected by quarantine during the COVID-19 epidemic in Southwestern China is higher than that among the unaffected population.^9^ A nationwide study also conducted by Seoul National University Bundang Hospital in South Korea concluded that the odds of COVID-19 survivors having depression were 3.34 higher than that of the control group.^15^ A cross-sectional study conducted in Jianghan Fangcang Shelter Hospital in China also concluded that the prevalence of anxiety and depressive symptoms in patients with COVID-19 were 18.6% and 13.4%, respectively.^16^ Furthermore, symptoms of depression were common among patients who had undergone quarantine for symptomatic and asymptomatic COVID-19 (44%), as highlighted by a study conducted in Jordan.^17^

In this cross-sectional study, we aimed to assess the prevalence of depression and anxiety among male patients with COVID-19 in LFH in Qatar and explore their relationship with participants’ demographic characteristics, perception of knowledge about COVID-19 and perceived health condition. To the best of our knowledge, this is the first study in Qatar looking at the prevalence of depression and anxiety in a field hospital dedicated to male migrant workers with COVID-19. This study can be a potential step to implement targeted interventions that help patients return to their everyday life.

## Methodology

### Study designs

This cross-sectional study was conducted between July 13, 2020 and August 23, 2020 among male patients with COVID-19 at LFH, a 504-bed facility opened by HMC in May 2020. Ethical approval was obtained from HMC Medical Research Center (Ref no. MRC-05-111).

We submitted the protocol on May 19, 2020, when Qatar's total number of COVID-19 cases was 33,969.

With Cochran's formula for sample size calculation, a minimum sample size of 380 was calculated based on a population size of 33,969, precision of 0.05 and a 95% confidence interval. For greater accuracy, we increased the sample size to 400.

A total of 400 patients were successfully recruited. Participants were given an information sheet about the study, written in their native language. Recruited patients consented to participate and complete the study anonymous paper surveys.

### Participants

All male patients with COVID-19, aged  ≥ 18 years, at LFH were given an information sheet one day before completing 14 days of their first positive swab PCR result. The study was explained to the patients in their language, and they had one day to decide regarding participation. Recruitment was finished after we reached a target sample of 400 consented participants.

We excluded patients who reported a previous history of a mental illness and patients who were unable to speak and read any of the languages in which the validated Patient Health Questionnaire-9 (PHQ-9) or Generalized Anxiety Disorder-7 (GAD-7) were available; i.e., English, Arabic, Hindi, Malayalam, Bangladeshi, and Urdu.^18-26^

### Instrument

We used a paper survey. To protect participants and team members, each participant was given an alcohol-sanitized pen, and the papers were kept in a closed drawer within a locked room for 14 days before data were entered into a password-locked computer for analysis. The survey consisted of three sections. The first is an introductory information section, asking about age, nationality, marital status, living condition, current job, monthly income, level of education, perceived knowledge about COVID-19, and perception of health condition.

The second section is the 9-item depression scale, PHQ-9.^18^ The third section is the 7-item anxiety scale, GAD-7.^19^ Each item in both scales is rated from 0 (not at all) to 3 (nearly every day) based on reported symptoms in the preceding 14 days. The survey was available in six different languages (English, Arabic, Hindi, Malayalam, Bangladeshi, and Urdu). The introductory information section was translated into these languages by bilingual healthcare professionals in HMC, and the validated translated versions of PHQ-9 and GAD-7 were utilized.^18–26^

PHQ-9 was used because of its ease of use, sensitivity to change over time, reliability and validity.^27^ We used the score cut-offs of 5, 10, 15, and 20 points to estimate the prevalence of mild, moderate, severe, and very severe depressive symptoms, respectively.^18^ GAD-7 scale was used as a brief screening tool for GAD that helps identify probable GAD cases and measure symptom severity. We used the score cut-offs of 5, 10, and 15 points to estimate the prevalence of mild, moderate, and severe anxiety symptoms, respectively.^19^

However, as the cut-off score of  ≥ 10 points was found to achieve the optimal balance between sensitivity and specificity for both PHQ-9 (88% and 85%, respectively) and GAD-7 (89% and 82%, respectively), this cut-off was used when conducting the association analysis.^28,29^

### Statistical analysis

Data were presented using descriptive statistics in the form of percentages for qualitative variables. A chi-square test was performed to compare the prevalence of depression, anxiety, and other variables. Logistic regression analysis was further undertaken to determine if any of the core demographic variables differed significantly in male patients with COVID-19 who were depressed and anxious. A one-sided *P*-value of  < 0.05 was considered significant. All statistical analyses were performed using SPSS for Windows (version 25.0; IBM Corp., Armonk, NY, USA).

## Results

### Descriptive data

The sample consisted of 400 participants (Table 1). Of all the subjects, 121 were  < 30 years old, 95 were 30–39 years old, 79 were 40–49 years old and 103 were 50 years or older.

The majority of participants were Nepalese (155), followed by Indians (71), Arabs (55), Bangladeshi (53) and Filipino (14), while other nationalities constituted 52 of the participants. Moreover, 295 were married, 101 were single, 3 were divorced, and 1 did not state his status (Table 1). Almost half of the participants (208) were living with colleagues, 130 were living alone, and 62 were living with their families. Most of the participants (324) had a full-time job, while 45 were nonemployed, 30 either had a part-time job or were retired, and 1 did not respond to this question. In addition, 160 participants had a monthly income of 5,000–15,000 QR, followed by 127 earning  < 2,000 QR, 101 earning 2,000–5,000 QR and 12 earning >15,000 QR. Then, 307 participants have a secondary school degree or higher; 349 participants considered themselves to have an average-to-good knowledge about COVID-19, 49 have poor knowledge about it and two did not answer this question. Furthermore, 394 participants perceived their health condition as average to good, while six perceived it as poor (Table 1).

### Main results

**Figure 1.**
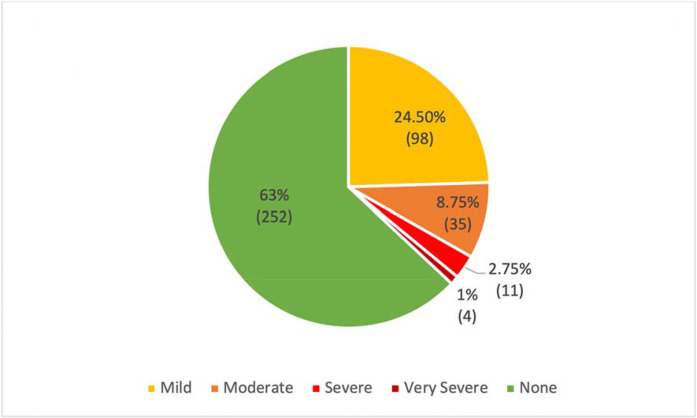
Prevalence of depressive symptoms among male patients with COVID-19 at Lebsayyer Field Hospital

**Figure 2.**
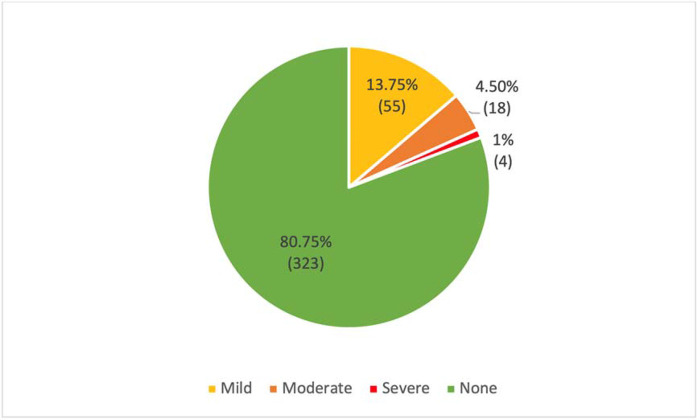
Prevalence of anxiety symptoms among male patients with COVID-19 at Lebsayyer Field Hospital

Of the 400 participants, 148 (37.0%) reported depressive symptoms (Table 2 and Figure 1), and 77 (19.3%) reported anxiety symptoms (Table 3 and Figure 2). The majority of the participants who reported depressive and anxiety symptoms had mild symptoms: 98 (24.5%) had PHQ-9 score of 5–9 points (depression) and 55 (13.8%) had GAD-7 score of 5–9 points (anxiety).

We based our analysis on those who reported at least moderate symptoms, and they were 50 (12.5%) participants scoring  ≥ 10 points in PHQ-9 (for depression) and 22 (5.5%) scoring  ≥ 10 points in GAD-7 (for anxiety). Interestingly, 72.7% of those with anxiety had comorbid depression simultaneously, i.e., 16 participants (4.0%) (Table 4).

Depression was three times more prevalent among those aged 40–49 years (20.3%) than those aged  < 30 years (6.6%) (*p* = 0.029). Bangladeshi participants had the highest prevalence of depression (26.4%), followed by Filipino participants (21.4%), while Arab participants had the least prevalence (7.3%) (*p* = 0.009). Although marital status had no significant effect on depression, those living with family had approximately twice more depression (22.6%) than those living alone (12.3%) or living with colleagues (9.6%) (*p* = 0.025). While employment status had no significant effect on depression, monthly income had a significant effect, with depression more prevalent among those getting the highest income of >15,000 QR (33.3%), followed by those who get the lowest salary of  < 2,000 QR (18.1%) (*p* = 0.004). The level of education and perception of knowledge about COVID-19 did not significantly affect the prevalence of depression. However, those who considered their health condition to be in the average range had a higher degree of depression (21.7%) (*p* = 0.035) (Table 4).

Anxiety was also most prevalent among Bangladeshi participants (15.1%), twice more common than in Filipino participants (7.1%) and five times more common than in Nepali participants (3.2%) who had the least prevalence of anxiety (*p* = 0.042). The participants with the lowest monthly income ( < 2,000 QR) had more anxiety than others (10.2%) (*p* = 0.039). Anxiety was most common among those who considered their health condition as poor (33.3%), three times more than those who considered their health condition as average (11.6%) and nine times more than those who considered their health condition as good (3.7%) (*p* = 0.001). Age group, marital status, living condition, employment status, education level, and perception of knowledge about COVID-19 had no significant effect on the prevalence of anxiety. None of the demographic variables had significant effects on the comorbid prevalence of depression and anxiety (Table 4).

The logistic regression model for depression was significant (*p*  <  0.05 on the omnibus tests of model coefficients and Hosmer and Lemeshow test). Using the forward stepwise logistic regression method, living condition, monthly income, education level, and perception of health condition were included in the final model. When considering each of the variables included in the analysis (Table 5), results revealed that living condition significantly predicted depressive symptoms in patients with COVID-19 admitted at LFH. In particular, those living with their family were more likely to be depressed. Monthly income, education level, and health perception did not significantly predict depression.

The logistic regression model for anxiety was significant (*p*  <  0.05 on the omnibus tests of model coefficients). Using the forward stepwise logistic regression method, perception of health condition was included in the final model. When considering the variable included in the analysis (Table 6), results showed that health perception significantly predicted anxiety symptoms in patients with COVID-19 admitted at LFH. Participants with poor health perception were more likely to be anxious.

While the models correctly classified 87.8% and 94.9% of the responses for depression and anxiety, respectively, there may be a range of variables beyond those captured in this study that may further explain why male patients with COVID-19 develop depression and anxiety.

## Discussion

To the best of our knowledge, this is the first study to explore depression and anxiety from a state-managed COVID-19 field hospital in Qatar. Although the distribution of nationalities was not entirely similar to that of Qatar's population, it was representative of patients at LFH, as all patients were invited to participate regardless of their nationality, and the survey was available in six languages. This study mainly found high levels of depressive and anxiety symptoms in men. The overall prevalence of depressive symptoms is found in 37.0% and 12.5% of the participants for a PHQ-9 cut-off of 5 and 10 points, respectively (Tables 2 and 4).^18^ Moreover, 19.3% and 5.5% of the respondents reported anxiety symptoms for a GAD-7 cut-off of 5 and 10 points, respectively (Tables 3 and 4).^19^ The prevalence of depressive symptoms in our study was lower than those in patients with SARS-CoV-1 infection during the SARS epidemic (50.6% using Beck Depression Inventory) and patients with COVID-19 in Wuhan (60.8% and 29.2% for a cut-off of 5 and 10 points, respectively, using PHQ-9).^30,31^ The social activities and privacy offered to patients at LFH and the mild COVID-19 infection of all patients can partially explain the lower prevalence.

Conversely, our study demonstrates higher rates of depression and anxiety than pre-pandemic rates of depression and anxiety among male Qatari nationals, who were surveyed using the Arabic version of the World Mental Health –Composite International Diagnostic Interview instrument. The major depression disorders were prevalent in 13.8% compared with 37.0% in our study, and generalized anxiety was found in 7.8% compared with 19.3% in our study.^32^ One possible explanation was that all our study participants were expatriates. The migrant population is well-documented to be more susceptible to stress; thus, mental health problems constitute a public-health problem worldwide.^33,34^

The prevalence of depressive symptoms in our study (37.0%) was similar to that of individuals within Qatar's institutional quarantine and isolation centers, which was 37.4% using a cut-off of 5 points on PHQ-9.^35^ By contrast, the prevalence of anxiety in the latter is greater than that of our study, that is, 25.9% using a cut-off of 5 points on GAD-7 compared with 19.3% in our study. This could be due to our inclusion of only male participants, as there might be increased fear related to COVID-19 among women, as shown in a cross-sectional study among Italian adults.^36^ Nonetheless, the former study had a very diverse sociodemographic sample that was not entirely comparable to our study sample. As expected, the psychological influence of the COVID-19 pandemic was higher among healthcare workers exposed to COVID-19 in Qatar than patients with COVID-19 at LFH, as 42.5% and 41.7% of medical residents exposed to COVID-19 reported depressive and anxiety symptoms (assessed by the Depression, Anxiety and Stress Scale: 21 Items) in a cross-sectional study at HMC.^37^ A possible explanation was that healthcare workers were at the forefront from the start of the COVID-19 pandemic, apart from their families, worried about themselves and their families from being infected along with the long working hours.

Among factors that might have altered the risk of depression in male patients with COVID-19 at LFH, recipients with the highest and lowest income reported more depressive symptoms (Table 4). In comparison, participants with the lowest income reported more anxiety symptoms. Unsteady family income has been associated with an increased risk of depression and anxiety during the pandemic.^38^ In this study, another associated factor was self-rated health. Anxiety was more common among those who had poor self-rated health (Table 6), which is consistent with previous studies.^39^ A third associated factor relates to living with a family instead of living alone or with colleagues. Those living with family had more depressive symptoms (Table 5), which is in accord with previous studies where patients with COVID-19 worry about family being infected.^40^ As regards ethnicity, Southeast Asians had the highest rates of depression and anxiety. This association is not consistent with a previous prevalence study in Qatar, which showed the lowest prevalence of major depressive episodes in participants from South Asia but the highest prevalence of subthreshold depressive episodes in Arabs and Southeast Asians.^41^ Contributing factors in the latter could be a chronic health condition, a variable not captured in our study, and psychosocial factors such as long working hours, physical demands of employment, and long-term separation from their families.^42,43^

## Limitations

Firstly, the most significant limitation in this study is the lack of female participants, as the hospital was allocated only for male patients. Secondly, the unbalanced nationality distribution, which reflects patients at LFH but not Qatar's population, could be due to a cluster of cases among Nepali workers who share housing. Thirdly, omitting the previous medical illnesses could be a confounding factor. Fourthly, we did not assess financial security, as employed participants were not asked if they were still being paid during their illness. Finally, the study is limited by its research design, which only gives a snapshot over a short period.

## Conclusion

The study data illustrate that the prevalence of depressive and anxiety symptoms is high among male patients who were hospitalized with noncritical COVID-19, and it is higher than the pre-pandemic reported rates in Qatar. The study identifies multiple associated factors. Firstly, the unsteady income, as the highest and lowest income, is associated with more depressive symptoms and the lowest income is associated with more anxiety symptoms. Secondly, poor self-rated health is associated with higher rates of anxiety symptoms. Thirdly, living with a family rather than with colleagues or living alone is associated with higher rates of depressive symptoms. Fourthly, Southeast Asian ethnicity has the highest rates of depression and anxiety. Finally, individuals aged 40–49 years have higher rates of depressive symptoms. The study also identifies significant predictive factors: living with a family was found to predict depressive symptoms, and poor health perception was found to predict anxiety symptoms.

### Authors' contributions

NZ: Study design, conduct of the study, collection and interpretation of data, statistical analysis, manuscript writing, and revision.

AA: Study design, conduct of the study, collection and interpretation of data, statistical analysis, manuscript writing, and revision

MA: Study design, conduct of the study, collection of data, and manuscript writing.

MM: Study design, conduct of the study, collection of data, and manuscript writing.

AM: Conduct of the study, collection of data, and manuscript writing.

SS: Conduct of the study, collection of data, and manuscript writing.

YN: Interpretation of data, and manuscript writing and revision.

MK: Interpretation of data, and manuscript writing and revision.

AA: Study design, conduct of the study, and manuscript revision.

All authors read and approved the final manuscript.

### Acknowledgments

We would like to thank all faculties in Family Medicine Residency Training Program in Qatar for their continuous guidance and support. Special thanks to Dr. Prem Chandra, Academic Research Scientist at HMC, for his outstanding efforts in data analysis. We extend our thanks to Dr. Sami Ouanes, Clinical Fellow in General Adult Psychiatry at HMC, for his great help in the interpretation of data.

### Competing interests

All authors have no competing interests.

### Funding sources

This research did not receive any specific grant from any funding agency.

## Figures and Tables

**Table 1:** Sociodemographic characteristics of the sample.

	Age					

Variables	< 30 years	30–39 years	40–49 years	≥ 50 years	Total	*P*-value

Nationality/Ethnicity						

Nepali	90 (74.4%)	38 (40%)	21 (26.6%)	5 (4.9%)	155 (38.8%)	0.001

Indian	1 (0.8%)	13 (13.7%)	25 (31.6%)	32 (31.1%)	71 (17.8%)	

Arab	17 (14%)	16 (16.8%)	7 (8.9%)	15 (14.6%)	55 (13.8%)	

Bangladeshi	6 (5%)	10 (10.5%)	13 (16.5%)	23 (22.3%)	53 (13.3%)	

Filipino	1 (0.8%)	3 (3.2%)	3 (3.8%)	7 (6.8%)	14 (3.5%)	

Others	6 (5%)	15 (15.8%)	10 (12.7%)	21 (20.4%)	52 (13%)	

Marital status						

Married	42 (35.1%)	81 (85.3%)	71 (89.9%)	100 (97.1%)	295 (73.9%)	0.001

Single	78 (64.9%)	13 (13.7%)	6 (7.6%)	3 (2.9%)	101 (25.4%)	

Divorced	0	1 (1.1%)	2 (2.5%)	0	3 (0.9%)	

Widower	0	0	0	0	0	

Living conditions						

With colleagues	72 (59.5%)	56 (58.9%)	49 (62%)	30 (29.1%)	208 (52%)	0.001

Alone	40 (33.1%)	26 (27.4%)	16 (20.3%)	48 (46.6%)	130 (32.5%)	

With family	9 (7.4%)	13 (13.7%)	14 (17.7%)	25 (24.3%)	62 (15.5%)	

Current job						

Nonemployed	12 (9.9%)	11 (11.6%)	10 (13%)	12 (11.7%)	45 (11.4%)	0.577

Employed part time	4 (3.3%)	8 (8.4%)	9 (11.7%)	5 (4.9%)	26 (6.6%)	

Employed full time	105 (86.8%)	74 (77.9%)	58 (73.7%)	85 (82.5%)	324 (81%)	

Retired	0	2 (2.1%)	1 (1.6%)	1 (1%)	4 (1%)	

Monthly income (QR)						

< 2,000	16 (13.2%)	26 (27.4%)	37 (46.8%)	47 (45.6%)	127 (31.8%)	0.001

2,000–5,000	23 (19.0%)	32 (33.7%)	18 (22.8%)	28 (27.2%)	101 (25.3%)	

5,000–15,000	80 (66.1%)	35 (36.8%)	20 (25.3%)	24 (23.3%)	160 (40%)	

> 15,000	2 (1.7%)	2 (2.1%)	4 (5.1%)	4 (3.9%)	12 (3%)	

Level of education						

No school	1 (0.8%)	2 (2.1%)	5 (6.3%)	8 (7.8%)	16 (4%)	0.001

Primary school	10 (8.3%)	16 (16.8%)	15 (19%)	35 (34%)	77 (19.3%)	

Secondary school	61 (50.4%)	45 (47.4%)	38 (48.1%)	41 (39.8%)	185 (46%)	

University or higher	49 (40.5%)	32 (33.7%)	21 (26.6%)	19 (18.4%)	122 (30.5%)	

How do you consider your knowledge about COVID-19?						

Poor	15 (12.4%)	14 (14.7%)	5 (6.3%)	15 (15%)	49 (12.4%)	0.001

Average	58 (47.9%)	38 (40%)	41 (51.9%)	44 (43%)	181 (45.5%)	

Good	48 (39.7%)	43 (45.3%)	33 (41.8%)	43 (42%)	168 (42.2%)	

How do you perceive your health condition?						

Poor	1 (0.8%)	4 (4.2%)	0	1 (1.0%)	6 (1.5%)	0.006

Average	13 (10.7%)	10 (10.5%)	21 (26.6%)	25 (24.3%)	69 (17.3%)	

Good	107 (88.4%)	81 (85.3%)	58 (73.4%)	77 (74.8%)	325 (81.3%)	

Total	121 (30.25%)	95 (23.75%)	79 (19.75%)	103 (25.75%)	400 (100%)	


**Table 2:** 

Depressive Symptoms	PHQ-9	Frequency	Percentage

Mild	5–9	98	(24.5%)

Moderate	10–14	35	(8.75%)

Severe	15–19	11	(2.75%)

Very severe	20–27	4	(1%)

Total		148	(37%)


**Table 3:** 

Anxiety Symptoms	GAD-7	Frequency	Percentage

Mild	5–9	55	(13.75%)

Moderate	10–14	18	(4.5%)

Severe	15–21	4	(1%)

Total		77	(19.25%)


**Table 4:** 

Variables	Depression	*P*-value	Anxiety	*P*-value	Depression and anxiety	*P*-value

Age						

< 30 years	8 (6.6%)	0.029	4 (3.3%)	0.456	3 (2.5%)	0.416

30–39 years	10 (10.5%)		4 (4.2%)		2 (2.1%)	

40–49 years	16 (20.3%)		5 (6.3%)		4 (5.1%)	

≥ 50 years	15 (14.6%)		8 (7.8%)		6 (5.8%)	

Nationality/ethnicity						

Bangladeshi	14 (26.4%)	0.009	8 (15.1%)	0.042	6 (11.3%)	0.106

Filipino	3 (21.4%)		1 (7.1%)		0 (0%)	

Indian	9 (12.7%)		3 (4.2%)		2 (2.8%)	

Nepali	12 (7.7%)		5 (3.2%)		4 (2.6%)	

Arab	4 (7.3%)		3 (5.5%)		2 (3.6%)

Others	8 (15.4%)		2 (3.8%)		2 (3.8%)	

Marital status						

Divorced	2 (66.7%)	0.134	2 (66.7%)	0.562	2 (66.7%)	0.326

Married	40 (13.6%)		16 (5.4%)		12 (4.1%)	

Single	8 (7.9%)		4 (4.0%)		2 (2%)	

Widower	0		0		0	

Living conditions						

With family	14 (22.6%)	0.025	5 (8.1%)	0.486	3 (4.8%)	0.935

Alone	16 (12.3%)		8 (6.2%)		5 (3.8%)	

With colleagues	20 (9.6%)		9 (4.3%)		8 (3.8%)	

Current job						

Nonemployed	6 (13.3%)	0.525	5 (11.1%)	0.181	4 (8.9%)	0.08

Employed part time	5 (19.2%)		1 (3.8%)		0 (0%)	

Employed full time	38 (11.7%)		15 (4.6%)		11 (3.4%)	

Retired	1 (25.0%)		1 (25.0%)		1 (25%)	

Monthly income (QR)						

< 2,000	23 (18.1%)	0.004	13 (10.2%)	0.039	10 (7.9%)	0.054

2,000–5,000	12 (11.9%)		4 (4.0%)		3 (3%)	

5,000–15,000	11 (6.9%)		5 (3.1%)		3 (1.9%)	

> 15,000	4 (33.3%)		0		0	

Level of education						

No school	3 (18.8%)	0.057	2 (12.5%)	0.328	1 (6.3%)	0.17

Primary school	12 (15.6%)		6 (7.8%)		5 (6.5%)	

Secondary school	28 (15.1%)		10 (5.4%)		9 (4.9%)	

University or higher	7 (5.7%)		4 (3.3%)		1 (0.8%)	

How do you consider your knowledge about COVID-19?						

Poor	4 (8.2%)	0.067	4 (8.2%)	0.234	4 (8.2%)	0.151

Average	23 (12.7%)		11 (6.1%)		6 (3.3%)	

Good	21 (12.5%)		5 (3.0%)		4 (2.4%)	

How do you perceive your health condition?						

Poor	1 (16.7%)	0.035	2 (33.3%)	0.001	1 (16.7%)	0.077

Average	15 (21.7%)		8 (11.6%)		5 (7.2%)	

Good	34 (10.5%)		12 (3.7%)		10 (3.1%)	

Total	50 (12.5%)	22 (5.5%)	16 (4%)	


Abbreviations: β, coefficients in the logistic regression equation; S.E., standard error of the coefficients; Wald, Wald statistic; eβ (odds ratio), proportionate change in odds.

**Table 5:** 

Predictor		β	S.E.	Wald	P-value	eβ (odds ratio)

Living condition	Living with colleagues vs. alone	− 0.24	0.39	0.39	0.53	0.79

	Living with family vs. alone	0.94	0.47	4.04	0.04	2.55

Monthly	2000–5000 vs. < 2000	− 0.09	0.42	0.05	0.83	0.91

Income (QR)	5000–15000 vs. < 2000	− 0.54	0.44	1.45	0.23	0.59

	>15000 vs. < 2000	1.53	0.80	3.64	0.06	4.63

Level of Education	Primary school vs. no school	− 0.57	0.77	0.55	0.46	0.56

	Secondary school vs. no school	0.05	0.74	0.00	0.95	1.05

	University or higher vs. No school	− 1.33	0.88	2.32	0.13	0.26

How do you	Average vs. poor	1.09	1.22	0.81	0.37	2.98

perceive your health condition?	Good vs. poor	0.05	1.19	0.00	0.97	1.05


Abbreviations: β, coefficients in the logistic regression equation; S.E., standard error of the coefficients; Wald, Wald statistic; eβ (odds ratio), proportionate change in odds.

**Table 6:** 

Predictor		β	S.E.	Wald	*P*-value	eβ (odds ratio)

How do you perceive	Average vs. poor	− 1.62	0.97	2.78	0.10	0.20

your health condition?	Good vs. poor	− 2.76	0.93	8.88	0.01	0.06


Abbreviations: β, coefficients in the logistic regression equation; S.E., standard error of the coefficients; Wald, Wald statistic; eβ (odds ratio), proportionate change in odds.
